# Measurable Residual Disease and Fusion Partner Independently Predict Survival and Relapse Risk in Childhood *KMT2A*-Rearranged Acute Myeloid Leukemia: A Study by the International Berlin-Frankfurt-Münster Study Group

**DOI:** 10.1200/JCO.22.02120

**Published:** 2023-03-30

**Authors:** Romy E. van Weelderen, Kim Klein, Christine J. Harrison, Yilin Jiang, Jonas Abrahamsson, Nira Arad-Cohen, Emmanuelle Bart-Delabesse, Barbara Buldini, Barbara De Moerloose, Michael N. Dworzak, Sarah Elitzur, José M. Fernández Navarro, Robert B. Gerbing, Bianca F. Goemans, Hester A. de Groot-Kruseman, Erin Guest, Shau-Yin Ha, Henrik Hasle, Charikleia Kelaidi, Hélène Lapillonne, Guy Leverger, Franco Locatelli, Riccardo Masetti, Takako Miyamura, Ulrika Norén-Nyström, Sophia Polychronopoulou, Mareike Rasche, Jeffrey E. Rubnitz, Jan Stary, Anne Tierens, Daisuke Tomizawa, C. Michel Zwaan, Gertjan J.L. Kaspers

**Affiliations:** ^1^ Princess Máxima Center for Pediatric Oncology, Utrecht, the Netherlands; ^2^ Pediatric Oncology, Emma Children's Hospital, Amsterdam UMC, Vrije Universiteit Amsterdam, Amsterdam, the Netherlands; ^3^ Wilhelmina Children's Hospital/University Medical Center Utrecht, Utrecht, the Netherlands; ^4^ Leukemia Research Cytogenetics Group, Translational and Clinical Research Institute, Newcastle University Centre for Cancer, Newcastle-upon-Tyne, United Kingdom; ^5^ Department of Pediatrics, Institute of Clinical Sciences, Salgrenska University Hospital, Gothenburg, Sweden; ^6^ Pediatric Hemato-Oncology Department, Ruth Rappaport Children's Hospital, Rambam Health Care Campus, Haifa, Israel; ^7^ IUC Toulouse-Oncopole, Laboratoire d’Hématologie secteur Génétique des Hémopathies, Toulouse, France; ^8^ Pediatric Hematology, Oncology and Stem Cell Transplant Division, Maternal and Child Health Department, Padua University, Padua, Italy; ^9^ Department of Pediatric Hematology-Oncology and Stem Cell Transplantation, Ghent University Hospital, Ghent, Belgium; ^10^ St. Anna Children's Hospital, Department of Pediatrics, Medical University of Vienna, and St Anna Children's Cancer Research Institute, Vienna, Austria; ^11^ Department of Pediatric Hematology and Oncology, Schneider Children's Medical Center and Tel Aviv University, Tel Aviv, Israel; ^12^ Pediatric Oncohematology Unit, Hospital Universitari i Politècnic la Fe, Valencia, Spain; ^13^ Department of Statistics, The Children's Oncology Group, Monrovia, California; ^14^ DCOG, Dutch Childhood Oncology Group, Utrecht, the Netherlands; ^15^ Children's Mercy Kansas City, Kansas City, MO; ^16^ Department of Pediatrics & Adolescent Medicine, Hong Kong Children's Hospital, Kowloon, Hong Kong; ^17^ Pediatrics and Adolescent Medicine, Aarhus University Hospital, Aarhus, Denmark; ^18^ Department of Pediatric Hematology and Oncology, Aghia Sophia Children's Hospital, Athens, Greece; ^19^ Pediatric Hematology and Oncology Department, Hôpital Armand Trousseau, Paris, France; ^20^ Department of Pediatric Hematology and Oncology and Cell and Gene Therapy, IRCCS Ospedale Pediatrico Bambino Gesù, Catholic University of the Sacred Heart, Rome, Italy; ^21^ Pediatric Oncology and Hematology, IRCCS Azienda Ospedaliero-Universitaria di Bologna, University of Bologna, Bologna, Italy; ^22^ Department of Pediatrics, Osaka University Graduate School of Medicine, Suita, Japan; ^23^ Department of Clinical Sciences, Pediatrics, Umeå University, Umeå, Sweden; ^24^ Department of Pediatric Hematology and Oncology, University Hospital Essen, Essen, Germany; ^25^ Department of Oncology, St Jude Children's Research Hospital, Memphis, TN; ^26^ Department of Pediatric Hematology and Oncology, University Hospital Motol and 2^nd^ Faculty of Medicine, Charles University, Prague, Czech Republic; ^27^ Department of Pathobiology and Laboratory Medicine, University Health Network, Toronto General Hospital, Toronto, ON, Canada; ^28^ Children's Cancer Center, National Center for Child Health and Development, Tokyo, Japan; ^29^ Department of Pediatric Oncology, Erasmus MC-Sophia Children's Hospital, Rotterdam, the Netherlands

## Abstract

A previous study by the International Berlin-Frankfurt-Münster Study Group (I-BFM-SG) on childhood *KMT2A*-rearranged (*KMT2A*-r) AML demonstrated the prognostic value of the fusion partner. This I-BFM-SG study investigated the value of flow cytometry-based measurable residual disease (flow-MRD) and evaluated the benefit of allogeneic stem-cell transplantation (allo-SCT) in first complete remission (CR1) in this disease.

**METHODS:**

A total of 1,130 children with *KMT2A*-r AML, diagnosed between January 2005 and December 2016, were assigned to high-risk (n = 402; 35.6%) or non–high-risk (n = 728; 64.4%) fusion partner-based groups. Flow-MRD levels at both end of induction 1 (EOI1) and 2 (EOI2) were available for 456 patients and were considered negative (<0.1%) or positive (≥0.1%). End points were 5-year event-free survival (EFS), cumulative incidence of relapse (CIR), and overall survival (OS).

**RESULTS:**

The high-risk group had inferior EFS (30.3% high risk *v* 54.0% non-high risk; *P* < .0001), CIR (59.7% *v* 35.2%; *P* < .0001), and OS (49.2% *v* 70.5%; *P* < .0001). EOI2 MRD negativity was associated with superior EFS (n = 413; 47.6% MRD negativity *v* n = 43; 16.3% MRD positivity; *P* < .0001) and OS (n = 413; 66.0% *v* n = 43; 27.9%; *P* < .0001), and showed a trend toward lower CIR (n = 392; 46.1% *v* n = 26; 65.4%; *P* = .016). Similar results were obtained for patients with EOI2 MRD negativity within both risk groups, except that within the non–high-risk group, CIR was comparable with that of patients with EOI2 MRD positivity. Allo-SCT in CR1 only reduced CIR (hazard ratio, 0.5 [95% CI, 0.4 to 0.8]; *P* = .00096) within the high-risk group but did not improve OS. In multivariable analyses, EOI2 MRD positivity and high-risk group were independently associated with inferior EFS, CIR, and OS.

**CONCLUSION:**

EOI2 flow-MRD is an independent prognostic factor and should be included as risk stratification factor in childhood *KMT2A*-r AML. Treatment approaches other than allo-SCT in CR1 are needed to improve prognosis.

## INTRODUCTION

Most pediatric AML study groups (SGs) currently base risk stratification on genetics, including fusion genes and molecular aberrations, and early treatment response, either morphologically or more accurately assessed by detection of flow cytometry-based measurable residual disease (flow-MRD).^1^ Detection of flow-MRD in bone marrow (BM) after induction therapy is considered a strong indicator of relapse,^2-7^ and can aid risk-directed postremission therapy,^2,5,6^ including allogeneic stem-cell transplantation (allo-SCT) in first complete remission (CR1).

**CONTEXT**

**Key Objective**
This study investigated whether flow cytometry-based measurable residual disease (flow-MRD) at end of induction 2 (EOI2) is of prognostic significance in childhood *KMT2A*-rearranged (*KMT2A*-r) AML. Moreover, the benefit of allogeneic stem-cell transplantation (allo-SCT) in first complete remission (CR1) was evaluated.
**Knowledge Generated**
To our knowledge, this study is the first to demonstrate that EOI2 flow-MRD positivity is an independent adverse prognosticator in childhood *KMT2A*-r AML, in addition to the high-risk *KMT2A* fusion partner-based group. Patients within the high-risk fusion partner-based group with EOI2 MRD positivity have dismal outcomes. Additionally, allo-SCT in CR1 reduces relapse risk in patients with high-risk *KMT2A* fusion partners.
**Relevance *(S. Bhatia)***
This study shows the utility of using EOI2 flow-MRD as an independent prognosticator for risk stratification in children with *KMT2A*-r AML, highlighting the need for novel therapeutic strategies for this subgroup of patients because of the dismal prognosis.*
*Relevance section written by *JCO* Associate Editor Smita Bhatia, MD, MPH, FASCO.

A heterogeneous genetic pediatric AML subtype for which international consensus on risk stratification is lacking^8^ is 11q23/*KMT2A*-rearranged (*KMT2A*-r) AML, which occurs in 20%-25% of children with AML.^1^ In a large International Berlin-Frankfurt-Münster (I-BFM)-SG collaborative study^9^ and in some smaller SG studies,^10,11^ the prognosis of this subtype has been shown to be influenced by the fusion partner. Considerably better survival rates have been reported for 1q21,^9^ whereas 6q27, 10p12, 10p11.2, 4q21, and 19p13.3 are considered high-risk *KMT2A* translocation partners, which independently predict poor prognosis.^9,10^ However, the prognostic value of flow-MRD in this specific disease is unknown and was questioned by the I-BFM-SG because of the relatively high number of relapses observed in patients with flow-MRD negativity (<0.1%).

The benefit of allo-SCT in CR1 remains a debatable subject in pediatric AML, as its enhanced antileukemic activity needs to outweigh the risk of transplant-related mortality.^1,12,13^ In previous pediatric *KMT2A*-r AML studies, allo-SCT in CR1 did not improve relapse risk, nor survival.^9,10^ However, exposure to gemtuzumab ozogamicin (GO; Mylotarg, Pfizer, New York, NY) before transplantation seemed to improve the post-transplantation prognosis in these patients.^10^

We aimed to evaluate the prognostic significance of end of induction 2 (EOI2) flow-MRD response and allo-SCT in CR1 in childhood *KMT2A*-r AML overall and within fusion partner-based risk groups.

## METHODS

### Study Design and Patients

A retrospective study was conducted within the I-BFM-SG, including 15 pediatric AML SGs/countries. Eligible patients were younger than 19 years and were newly diagnosed with *KMT2A*-r AML between January 1, 2005, and December 31, 2016. Patients with a diagnosis of myeloid leukemia in Down syndrome, isolated myeloid sarcoma, or acute promyelocytic leukemia were excluded, as well as patients who were initially treated, for more than one week, for a diagnosis other than AML. Not all SGs/countries provided eligible patients for the entire study period, and treatment was given according to national or international pediatric AML SG trials (Data Supplement [Table S1], online only), which were all cytarabine-/anthracycline-based.^2,4,6,14-24^ Institutional ethics committees approved these trials, and patients and/or parents provided written consent according to the Declaration of Helsinki. All data were checked for accuracy and corrected in consultation with the SGs/countries.

### Assignment to Fusion Partner Groups and Risk Classification

The karyotype (G-, Q-, or R-banding), fluorescence in situ hybridization, and reverse transcription polymerase chain reaction (PCR) results were reviewed within the SGs/countries for the presence of *KMT2A* rearrangements. Patients were assigned to 10 different fusion partner groups or the *KMT2A*-other group, as previously reported by Balgobind et al.^9^ The group assignment was reviewed by two authors (R.W. and C.H.). The fusion partners of *KMT2A*-other group patients were reviewed to identify novel groups, with a minimum of 10 patients. Patients with unidentified fusion partners or those occurring in less than 10 patients remained within the *KMT2A*-other group.

On the basis of previously published classifications,^9,10^ patients with 6q27 (*KMT2A::AFDN*), 4q21 (*KMT2A::AFF1*), 10p12 (*KMT2A::MLLT10*), 10p11.2 (*KMT2A::ABI1*), and 19p13.3 (*KMT2A::MLLT1*) were assigned to the high-risk group, while all others were assigned to the non–high-risk group. The *KMT2A*-other group was excluded because their fusion partners could not be risk group–assigned owing to their unknown prognostic impact.

### Flow-MRD Analysis

Flow-MRD analysis was implemented in most, but not yet in all, trials/treatment protocols used by the SGs/countries during the study period. Additionally, some SGs/countries could not provide flow-MRD data because of ongoing trials. Ten SGs/countries provided flow-MRD data, which were mainly detected using 4- to 10-color antibody panels. Two SGs also used the different from normal approach. Details on the BM cellularity were not collected.

### Definitions and Statistical Analysis

CR was defined as <5% BM blasts, the absence of cells with Auer rods and extramedullary disease, and peripheral blood cell regeneration.^1^ Refractory disease was defined as ≥5% BM blasts, either morphologically, cytogenetically, or by a high positive PCR result, or proven extramedullary disease after induction therapy. Relapse was defined as ≥5% BM blasts or reappearance of blasts in peripheral blood, or the development of extramedullary disease after initial morphologic CR.^1^ End of induction 1 (EOI1) and EOI2 BM responses were morphologically categorized as M1 (<5% blasts), M2 (≥5%-<20% blasts), or M3 (≥20% blasts). EOI1 and EOI2 flow-MRD responses <0.1% were considered negative and ≥0.1% positive. Event-free survival (EFS) was defined as the time from diagnosis to induction failure, relapse, secondary malignancy, death in CR, or last follow-up, whichever occurred first. Induction failure was included as an event at t = 0. Cumulative incidence of relapse (CIR) and nonrelapse mortality (NRM) were defined as the time from EOI1, for patients in CR, to relapse and to death without relapse, respectively. The competing event for CIR was death without relapse and for NRM death with relapse. Overall survival (OS) was defined as the time from diagnosis to death, or last follow-up.

Median (IQR) follow-up time of patients was 5.2 years (3.5-7.8). Differences in proportions were tested using Pearson's χ^2^ test. Medians between two groups were compared using the Mann-Whitney *U* test. EFS and OS estimates were calculated using the Kaplan-Meier method and compared using the log-rank test. The CIR was estimated by adjusting for competing risks and was compared using Gray's test. Hazard ratios (HR) were calculated using Cox proportional hazard models, wherein allo-SCT in CR1 was included as a time-dependent covariate. Covariates with two-sided *P* values < .05 in univariable analysis were included in multivariable analysis. To visually compare the CIR, NRM, and OS of allo-SCT versus no allo-SCT in CR1 overall and stratified by treatment era (2005-2010 *v* 2011-2016), a 90-day landmark was used. Multivariable analyses were performed excluding morphologic BM and flow-MRD response, referred to as the crude models containing the highest number of patients, and including morphologic BM and flow-MRD response separately from each other. Analyses were performed using R version 4.0.3. Two-sided *P* values ≤ .01 were considered statistically significant.

## RESULTS

### Patients

**FIG 1.**
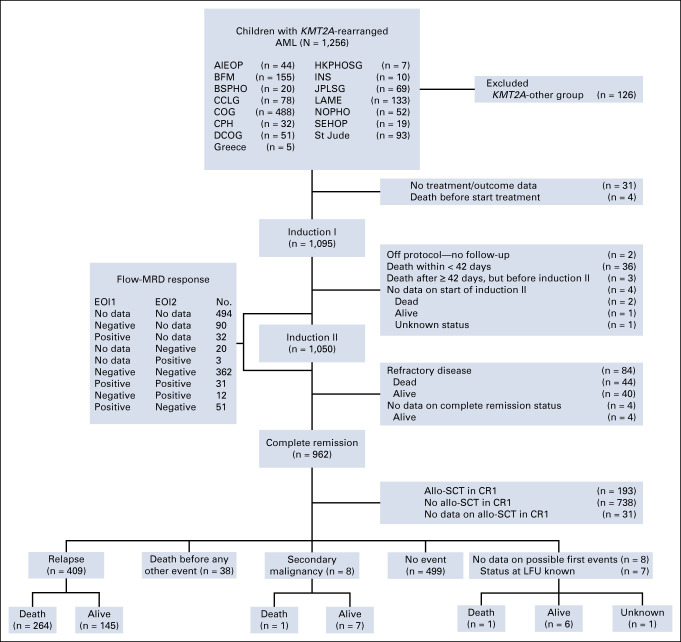
Flow diagram of the cohort of children with *KMT2A*-rearranged AML recruited by the collaborative study groups/countries between January 1, 2005, and December 31, 2016. Patients with unidentified fusion partners or those occurring in less than 10 patients were assigned to the *KMT2A*-other group. Fusion partners in the *KMT2A*-other group could not be risk group-assigned because of their unknown prognostic impact and were excluded. AIEOP, Associazione Italiana Ematologia Oncologia Pediatrica (Italy); allo-SCT, allogeneic stem-cell transplantation; BFM, Berlin-Frankfurt-Münster (Germany and Austria); BSPHO, Belgian Society of Pediatric Hematology Oncology (Belgium); CCLG, Children's Cancer and Leukaemia Group (United Kingdom); COG, Children's Oncology Group (United States); CPH, Czech Pediatric Hematology (Czech Republic); CR1, first complete remission; DCOG, Dutch Childhood Oncology Group (the Netherlands); EOI1, end of induction 1; EOI2, end of induction 2; HKPHOSG, Hong Kong Pediatric Hematology and Oncology Study Group (Hong Kong); I-BFM, International Berlin-Frankfurt-Münster; INS, Israel National Study (Israel); JPLSG, Japanese Pediatric Leukemia/Lymphoma Study Group (Japan); LAME, Leucémie Aiguë Myéloblastique Enfant (France); LFU, last follow-up; MRD, measurable residual disease; No., number of patients; NOPHO, Nordic Society for Pediatric Hematology and Oncology (Scandinavia); SEHOP, Spanish Society of Pediatric Hematology and Oncology (Spain); St Jude, St Jude Children's Research Hospital (United States).

Of the 1,256 eligible patients, 126 (10.0%) were assigned to the *KMT2A*-other group and excluded (Fig 1). *KMT2A*-other group patients were younger and had more often a WBC at diagnosis ≥100 × 10^9^/L than the remaining 1,130 patients (Data Supplement, Tables S2 and S3). Table 1 presents the clinical characteristics of these 1,130 patients and stratified by *KMT2A* risk group (non-high risk: n *=* 728, 64.4%; high risk: n *=* 402, 35.6%). High-risk group patients tended to be more often age 10 years and older (30.1% high-risk group *v* 23.4% non–high-risk group; *P* = .013), had a higher median WBC at diagnosis (25.7 × 10^9^/L *v* 16.2 × 10^9^/L; *P* = .0056), and had less frequently good morphologic EOI1 BM responses (ie, M1; 71.0% *v* 83.8%; *P* < .0001), with a trend for less frequent EOI2 M1 BM responses (91.9% *v* 95.6%; *P* = .015).

**TABLE 1.**
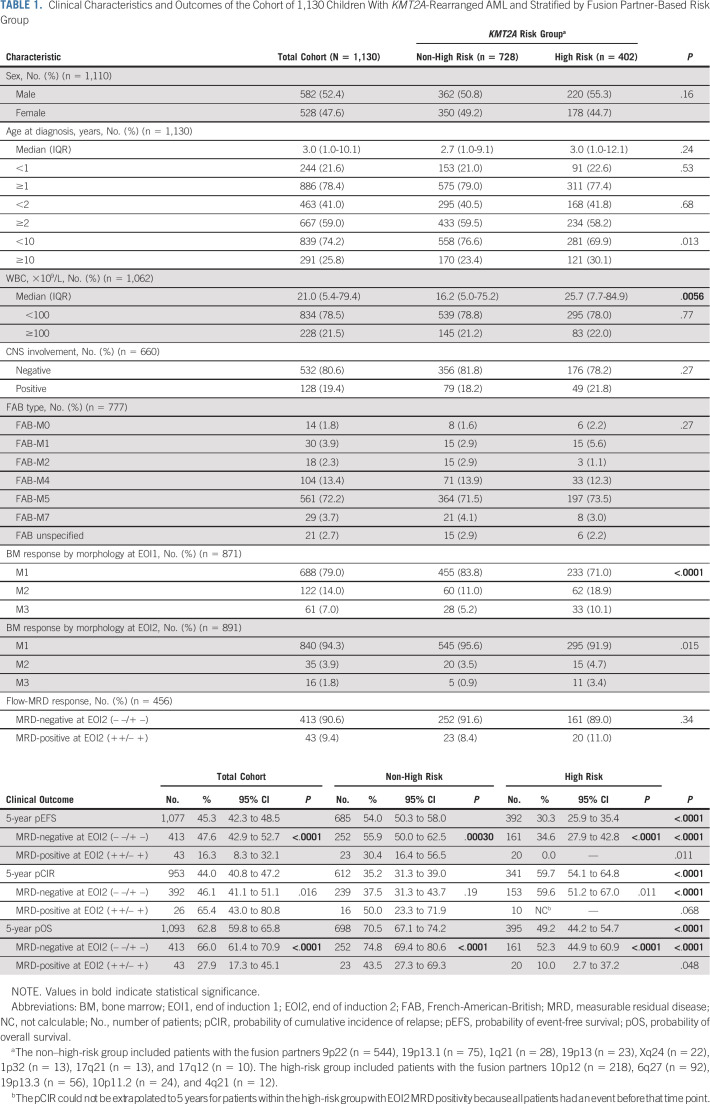
Clinical Characteristics and Outcomes of the Cohort of 1,130 Children With *KMT2A*-Rearranged AML and Stratified by Fusion Partner-Based Risk Group

Of the 1,130 included patients, 456 (40.4%) had available EOI1 and EOI2 MRD data (Fig 1). No significant differences in clinical characteristics and outcomes were detected between patients with and without available MRD data at both time points (Data Supplement, Tables S4 and S5). These 456 patients were classified as either EOI2 MRD-negative (n *=* 413; 90.6%), including patients with negative-negative (– –; n *=* 362; 79.4%) and positive-negative (+ –; n *=* 51; 11.2%) MRD responses at the respective time points, or EOI2 MRD-positive (n *=* 43; 9.4%), including patients with positive-positive (+ +; n *=* 31; 6.8%) and negative-positive (– +; n *=* 12; 2.6%; see Data Supplement, Table S6, for characteristics) MRD responses. The proportions of patients with EOI2 MRD negativity and MRD positivity were similar within both *KMT2A* risk groups (Table 1).

Data on allo-SCT in CR1 were available for 931/962 (96.8%) patients who achieved CR, of whom 20.7% (193/931) underwent transplantation. The Data Supplement, Table S7, details the transplantation characteristics. The transplantation rates within the non–high-risk and high-risk groups were 17.0% (103/605) and 27.6% (90/326), respectively. Pre-SCT MRD data were available for 68 patients, of whom eight were MRD-positive and 60 MRD-negative.

### Outcome in Childhood *KMT2A*-r AML and Prognostic Significance of EOI2 Flow-MRD Response

Of the 1,095 patients who were known to have started chemotherapy, 962 (87.9%) achieved CR. The cohort's 5-year EFS, CIR, and OS rates were 45.3% (95% CI, 42.3 to 48.5), 44.0% (40.8-47.2), and 62.8% (59.8-65.8), respectively (Table 1). The high-risk group had inferior EFS (30.3% [95% CI, 25.9 to 35.4] *v* 54.0% [95% CI, 50.3 to 58.0]; *P* < .0001), CIR (59.7% [95% CI, 54.1 to 64.8] *v* 35.2% [95% CI, 31.3 to 39.0]; *P* < .0001), and OS (49.2% [95% CI, 44.2 to 54.7] *v* 70.5% [95% CI, 67.1 to 74.2]; *P* < .0001) than the non–high-risk group.

**FIG 2.**
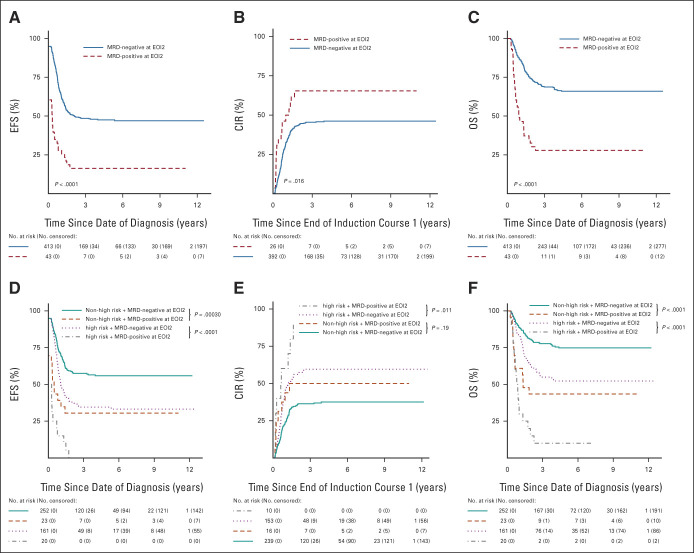
(A) EFS, (B) CIR, and (C) OS curves of patients with EOI2 MRD negativity and MRD positivity. (D) EFS, (E) CIR, and (F) OS curves of patients with EOI2 MRD negativity and MRD positivity stratified by *KMT2A* fusion partner-based risk group. The group of patients with EOI2 MRD negativity included patients who were EOI1 MRD-negative and EOI2 MRD-negative (– –), as well as patients who were EOI1 MRD-positive and EOI2 MRD-negative (+ –). The group of patients with EOI2 MRD positivity included patients who were EOI1 MRD-positive and EOI2 MRD-positive (+ +), as well as patients who were EOI1 MRD-negative and EOI2 MRD-positive (– +). CIR, cumulative incidence of relapse; EFS, event-free survival; EOI1, end of induction 1; EOI2, end of induction 2; MRD, measurable residual disease; OS, overall survival.

Patients with EOI2 MRD negativity had better EFS (n *=* 413; 47.6% [95% CI, 42.9 to 52.7] *v* n *=* 43; 16.3% [95% CI, 8.3 to 32.1]; *P* < .0001) and OS (n *=* 413; 66.0% [95% CI, 61.4 to 70.9] *v* n *=* 43; 27.9% [95% CI, 17.3 to 45.1]; *P* < .0001) than patients with EOI2 MRD positivity, and showed a trend toward lower CIR (n *=* 392; 46.1% [95% CI, 41.1 to 51.1] *v* n *=* 26; 65.4% [95% CI, 43.0 to 80.8]; *P* = .016; Table 1; Figs 2A-2C). Similarly, within both *KMT2A* risk groups, patients with EOI2 MRD negativity had significantly better EFS and OS than patients with EOI2 MRD positivity (Table 1; Figs 2D and 2F). With regard to CIR, within the non–high-risk group, patients with EOI2 MRD negativity and MRD positivity had similar CIR (n *=* 239; 37.5% [95% CI, 31.3 to 43.7] *v* n *=* 16; 50.0% [95% CI, 23.3 to 71.9]; *P* = .19), whereas within the high-risk group, patients with EOI2 MRD negativity showed a trend toward lower CIR (n *=* 153; 59.6% [95% CI, 51.2 to 67.0] *v* n *=* 10, 5-year CIR not calculable because all events occurred before this time point; *P* = .011; Table 1; Fig 2E).

Conversely, among patients with EOI2 MRD negativity, the high-risk group had inferior EFS (n *=* 161; 34.6% [95% CI, 27.9 to 42.8] *v* n *=* 252; 55.9% [95% CI, 50.0 to 62.5]), CIR (n *=* 153; 59.6% [95% CI, 51.2 to 67.0] *v* n *=* 239; 37.5% [95% CI, 31.3 to 43.7]), and OS (n *=* 161; 52.3% [95% CI, 44.9 to 60.9] *v* n *=* 252; 74.8% [95% CI, 69.4 to 80.6]) than the non–high-risk group (all *P* < .0001; Table 1). Among patients with EOI2 MRD positivity the high-risk group showed a trend toward inferior EFS (n *=* 20; 0.0% *v* n *=* 23; 30.4% [95% CI, 16.4 to 56.5]; *P* = .011), whereas CIR and OS were similar for the *KMT2A* risk groups (Table 1).

### Univariable and Multivariable Analyses

In univariable analyses (Data Supplement, Table S8), EOI2 MRD positivity and the high-risk group were significantly associated with inferior EFS, CIR, and OS. Age 2 years and older and age 10 years and older were significantly associated with inferior OS. WBC ≥100 × 10^9^/L showed a trend toward inferior EFS and was significantly associated with poorer OS. EOI1 M2 and M3 BM responses were significantly associated with inferior EFS. Additionally, EOI1 and EOI2 M3 BM responses were significantly associated with inferior OS.

**FIG 3.**
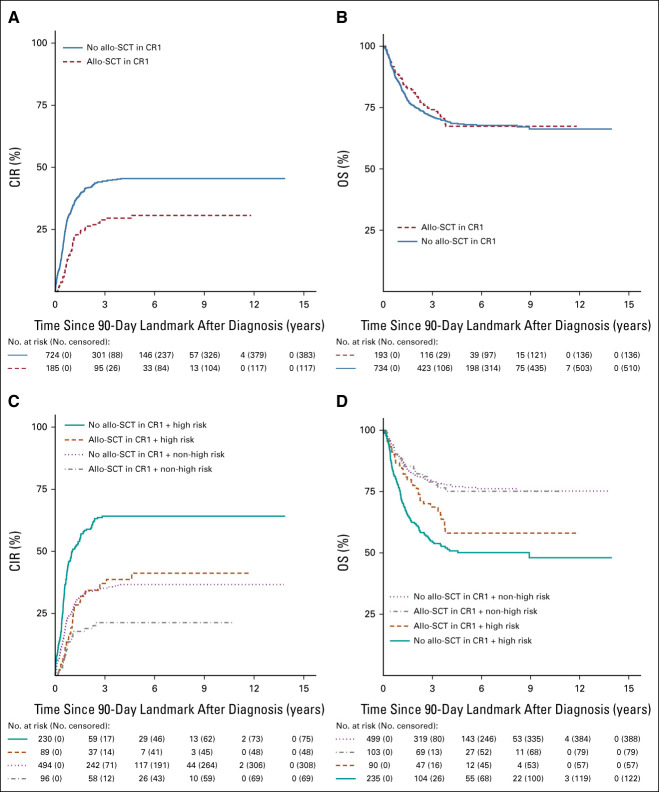
(A) CIR and (B) OS curves to visually compare (with a 90-day landmark) patients who did and did not receive allo-SCT in CR1. (C) CIR and (D) OS curves to visually compare (with a 90-day landmark) patients who did and did not receive allo-SCT in CR1 stratified by *KMT2A* fusion partner-based risk group. *P* values are not shown as these figures are only used for visual comparison. allo-SCT, allogeneic stem-cell transplantation; CIR, cumulative incidence of relapse; CR1, first complete remission; OS, overall survival.

Overall, allo-SCT in CR1 showed a trend toward decreased CIR (HR 0.7 [95% CI, 0.5 to 0.9]; *P* = .011) but did not improve OS (HR 1.0 [95% CI, 0.7 to 1.3]; *P* = .99; Figs 3A and 3B for 90-day landmark), nor within either *KMT2A* risk group. Within the high-risk group, allo-SCT in CR1 decreased CIR (HR 0.5 [95% CI, 0.4 to 0.8]; *P* = .00096), but not within the non-high-risk group (HR 0.6 [95% CI, 0.4 to 1.0]; *P* = .058; Figs 3C and 3D for 90-day landmark). Within smaller groups on the basis of *KMT2A* risk group and EOI2 MRD response, allo-SCT in CR1 did not significantly decrease CIR or improve OS (Data Supplement, Table S8). The Data Supplement, Figures S1 and S2, present 90-day NRM landmark analyses for allo-SCT versus no allo-SCT in CR1 and 90-day NRM, CIR, and OS landmark analyses for these patients stratified by treatment era (2005-2010 *v* 2011-2016).

The Data Supplement, Table S9, presents the crude multivariable models and those including morphologic BM response. In multivariable analyses including flow-MRD response (Table 2), EOI2 MRD positivity and the high-risk group were independently associated with inferior EFS (HR 3.4 [95% CI, 2.4 to 4.9]; *P* < .0001; HR 2.0 [95% CI, 1.5 to 2.5]; *P* < .0001, respectively), CIR (HR 2.3 [95% CI, 1.4 to 3.7]; *P* = .0013; HR 2.1 [95% CI, 1.5 to 2.8]; *P* < .0001, respectively), and OS (HR 1.9 [95% CI, 1.6 to 2.3]; *P* < .0001; HR 2.1 [95% CI, 1.5 to 2.9]; *P* < .0001, respectively). Age 10 years and older was independently associated with poorer OS (HR 1.7 [95% CI, 1.2 to 2.3]; *P* = .0023).

**TABLE 2.**
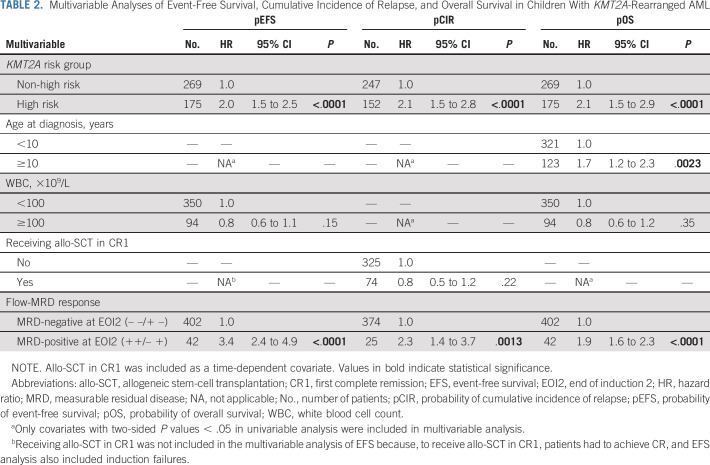
Multivariable Analyses of Event-Free Survival, Cumulative Incidence of Relapse, and Overall Survival in Children With *KMT2A*-Rearranged AML

## DISCUSSION

After our previous I-BFM-SG study showing the prognostic value of the *KMT2A* fusion partner,^9^ this novel study on childhood *KMT2A*-r AML is, to our knowledge, the first to demonstrate that EOI2 MRD positivity is an independent adverse prognostic factor in this disease, in addition to the fusion partner. This further demonstrates the need for risk stratification in childhood *KMT2A*-r AML, which constitutes 20%-25% of pediatric AML cases. In addition, allo-SCT in CR1 reduced relapse risk within the high-risk group but did not improve OS, as it was counterbalanced by enhanced procedure-related toxicity.

We confirmed the independent adverse prognostic significance of previously defined high-risk *KMT2A* translocation partners (ie, 4q21, 6q27, 10p11.2, 10p12, and 19p13.3).^9,10^ Therefore, our study serves as a consensus statement on fusion partner-based risk classification of childhood *KMT2A*-r AML, which will enable intergroup clinical trials and facilitate the performance of retrospective collaborative studies. The Children's Oncology Group (COG), as well as other SGs, incorporated these high-risk translocations into the initial risk stratification of their ongoing AAML1831 trial (ClinicalTrials.gov identifier: NCT04293562).^25,26^ Hopefully, more SGs will follow.

Interestingly, the EFS and OS rates of patients within the non–high-risk group with EOI2 MRD positivity were quite similar to those of patients within the high-risk group with EOI2 MRD negativity (EFS, 30.4% *v* 34.6%; OS, 43.5% *v* 52.3%). Notably, despite their good initial treatment response, the CIR rate of patients with EOI2 MRD negativity was 46%, which is markedly higher than the reported CIR rates of 17% and 32% of patients with EOI2 MRD negativity in pediatric AML in general.^2,5^ This finding demonstrates the aggressive nature of childhood *KMT2A*-r AML. Relapses may be inherent to *KMT2A* genetic features, causing the emergence of leukemic stem cells not detected by flow-MRD after killing the leukemic cell bulk.^27^ Leukemic stem-cell frequency assessment at diagnosis and during treatment may be included in future studies to further improve the identification of children with *KMT2A*-r AML at risk of relapse.^28,29^ Alternatively, the limited sensitivity of the 4- to 10-color antibody panels used for flow-MRD detection in our study may have played a role. It needs to be determined whether flow-MRD detection on the basis of up-to-date methodology with 10 or more color approaches and centralized quality control,^30,31^ PCR-based techniques, or next-generation sequencing (NGS) may at least partially overcome this limitation in sensitivity.

Among patients with EOI2 MRD negativity, the high-risk group showed significantly poorer outcomes than the non–high-risk group, consistent with a previous study on children with AML with MRD negativity that identified t(6;11) and t(10;11)—breakpoints that were not further specified—as independent adverse prognostic factors.^32^ By contrast, among patients with EOI2 MRD positivity, prognosis was not significantly influenced by the *KMT2A* risk group.

On the basis of our findings, patients within the high-risk group, irrespective of flow-MRD response, and patients within the non–high-risk group with EOI2 MRD positivity may benefit from high-risk–based treatment or novel treatment approaches, including experimental therapy. Allo-SCT in CR1 is generally used as high-risk–based treatment. The allo-SCT in CR1 rate in our study was slightly higher than in the previous I-BFM-SG study^9^ (21% v 14%) but relatively low by modern standards, and given the high relapse risk of children with *KMT2A*-r AML. This may be explained by the fact that in the treatment protocols used during our study period, high-risk *KMT2A* translocations were not always considered a transplantation indication or that transplantation was restricted to those with available human leukocyte antigen–matched donors. To our knowledge, we are the first to show that allo-SCT in CR1 reduced the relapse risk in patients within the high-risk group, but not in patients within the non–high-risk group, nor within smaller groups on the basis of *KMT2A* risk group and EOI2 MRD response. This should be interpreted cautiously, as the overall transplantation rate was low and this study was not powered to assess the effect of allo-SCT in CR1. Furthermore, allo-SCT in CR1 did not improve OS, consistent with previous findings.^9,10^ This is most likely due to insufficient eradication of the disease and, although significantly reduced in the most recent years, transplantation-related mortality.

Our results highlight the need for new treatment approaches to improve the prognosis of children with *KMT2A*-r AML. The COG demonstrated that GO added to induction therapy improved EFS and reduced relapse risk in these patients.^10^ Additionally, GO seemed to improve post-transplantation prognosis.^10^ These results need to be further confirmed to definitively establish which children with *KMT2A*-r AML show the greatest benefit from intensified treatment strategies. In the current MyeChild01 (ClinicalTrials.gov identifier: NCT02724163) and Japanese Pediatric Leukemia/Lymphoma SG-AML-20 (jRCTs041210015) trials,^33^ GO added to induction and postinduction therapy is being studied, respectively. Other potentially promising agents are menin inhibitors, which have shown a significant reduction in leukemic cell load in mice engrafted with *KMT2A*-r AML.^34^ Additionally, in two recent phase 1 studies, treatment with the menin inhibitors SNDX-5613 (revumenib)^35^ and KO-539 (ziftomenib)^36^ appeared safe and showed encouraging clinical responses in patients with relapsed/refractory *KMT2A*-r acute leukemia and AML, respectively.

Although flow cytometry is applicable in approximately 90% of children with AML, MRD cannot be detected in all patients because it requires extensive expertise to distinguish AML cells from normal, regenerating BM, which may only be available in larger experienced laboratories.^5,30,37^ Considerable efforts are being made to standardize sample preparation and analyses.^5,30^

This largest cohort of children with *KMT2A*-r AML serves as a highly valuable historical cohort for future pediatric AML SG collaborations in the pre-menin inhibitor era. Our study was limited by its retrospective design, the use of nonuniform treatment protocols with different risk stratifications across SGs, the overall low transplantation rate, and the lack of flow-MRD data at both time points in more than half of the patients. In some cases, MRD availability might have interacted with risk stratification and/or treatment allocation, including allo-SCT in CR1. Nonetheless, the relatively old and largely nonstandardized flow-MRD data from our cohort allowed us to discern flow-MRD–based risk associations independent of the fusion partner-based risk group.

We conclude that EOI2 flow-MRD response and fusion partner-based risk group should be included as risk stratification factors in childhood *KMT2A*-r AML. Patients within the non–high-risk group with EOI2 MRD negativity may be assigned to the standard-risk arm of treatment protocols but should be closely monitored for MRD after remission. All other patients (Data Supplement, Figure S3) should be assigned to the high-risk arm. Future studies should establish whether allo-SCT in CR1 will be the best risk-adapted treatment for all patients assigned to the high-risk-arm. New treatment approaches, including GO and menin inhibitors, are urgently needed for this subset of patients. The implementation of optimized stratification approaches, the increased availability of flow-MRD assays, quantitative PCR, and NGS among pediatric AML SGs worldwide, and the use of agents able to kill *KMT2A*-r AML cells more efficiently could contribute to improved survival of children with *KMT2A*-r AML, for whom international collaborative research remains indispensable.

## Data Availability

Individual participant data are not available to share. Participating study groups should be contacted directly for the original data.

## References

[1] Creutzig U van den Heuvel-Eibrink MM Gibson B : Diagnosis and management of acute myeloid leukemia in children and adolescents: Recommendations from an international expert panel. Blood 120:3187-3205, 2012 22879540 10.1182/blood-2012-03-362608

[2] Rubnitz JE Inaba H Dahl G : Minimal residual disease-directed therapy for childhood acute myeloid leukaemia: Results of the AML02 multicentre trial. Lancet Oncol 11:543-552 20451454 10.1016/S1470-2045(10)70090-5 PMC3171799

[3] Segerink WH de Haas V Kaspers GJL: Measurable residual disease in pediatric acute myeloid leukemia: A systematic review. Expert Rev Anticancer Ther 21:451-459, 2021 33706635 10.1080/14737140.2021.1860763

[4] Rubnitz JE Lacayo NJ Inaba H : Clofarabine can replace anthracyclines and etoposide in remission induction therapy for childhood acute myeloid leukemia: The AML08 multicenter, randomized phase III trial. J Clin Oncol 37:2072-2081, 2019 31246522 10.1200/JCO.19.00327 PMC7001777

[5] Buldini B Rizzati F Masetti R : Prognostic significance of flow-cytometry evaluation of minimal residual disease in children with acute myeloid leukaemia treated according to the AIEOP-AML 2002/01 study protocol. Br J Haematol 177:116-126, 2017 28240765 10.1111/bjh.14523

[6] Tierens A Bjorklund E Siitonen S : Residual disease detected by flow cytometry is an independent predictor of survival in childhood acute myeloid leukaemia; results of the NOPHO-AML 2004 study. Br J Haematol 174:600-609, 2016 27072379 10.1111/bjh.14093

[7] Inaba H Coustan-Smith E Cao X : Comparative analysis of different approaches to measure treatment response in acute myeloid leukemia. J Clin Oncol 30:3625-3632, 2012 22965955 10.1200/JCO.2011.41.5323 PMC3462046

[8] Klein K de Haas V Kaspers GJL: Clinical challenges in de novo pediatric acute myeloid leukemia. Expert Rev Anticancer Ther 18:277-293, 2018 29338495 10.1080/14737140.2018.1428091

[9] Balgobind BV Raimondi SC Harbott J : Novel prognostic subgroups in childhood 11q23/MLL-rearranged acute myeloid leukemia: Results of an international retrospective study. Blood 114:2489-2496, 2009 19528532 10.1182/blood-2009-04-215152 PMC2927031

[10] Pollard JA Guest E Alonzo TA : Gemtuzumab ozogamicin improves event-free survival and reduces relapse in pediatric KMT2A-rearranged AML: Results from the phase III Children's Oncology Group trial AAML0531. J Clin Oncol 39:3149-3160, 2021 34048275 10.1200/JCO.20.03048 PMC8478392

[11] Pigazzi M Masetti R Bresolin S : MLL partner genes drive distinct gene expression profiles and genomic alterations in pediatric acute myeloid leukemia: An AIEOP study. Leukemia 25:560-563, 2011 21331072 10.1038/leu.2010.316

[12] Horan JT Alonzo TA Lyman GH : Impact of disease risk on efficacy of matched related bone marrow transplantation for pediatric acute myeloid leukemia: The Children's Oncology Group. J Clin Oncol 26:5797-5801, 2008 18955460 10.1200/JCO.2007.13.5244 PMC2645105

[13] Niewerth D Creutzig U Bierings MB : A review on allogeneic stem cell transplantation for newly diagnosed pediatric acute myeloid leukemia. Blood 116:2205-2214, 2010 20538803 10.1182/blood-2010-01-261800

[14] Pession A Masetti R Rizzari C : Results of the AIEOP AML 2002/01 multicenter prospective trial for the treatment of children with acute myeloid leukemia. Blood 122:170-178, 2013 23673857 10.1182/blood-2013-03-491621

[15] Creutzig U Zimmermann M Bourquin JP : Randomized trial comparing liposomal daunorubicin with idarubicin as induction for pediatric acute myeloid leukemia: Results from study AML-BFM 2004. Blood 122:37-43, 2013 23704089 10.1182/blood-2013-02-484097

[16] Moerloose B Reedijk A Bock GH : Response-guided chemotherapy for pediatric acute myeloid leukemia without hematopoietic stem cell transplantation in first complete remission: Results from protocol DB AML-01. Pediatr Blood Cancer 66:e27605, 2019 30623572 10.1002/pbc.27605

[17] Gamis AS Alonzo TA Meshinchi S : Gemtuzumab ozogamicin in children and adolescents with de novo acute myeloid leukemia improves event-free survival by reducing relapse risk: Results from the randomized phase III Children's Oncology Group trial AAML0531. J Clin Oncol 32:3021-3032, 2014 25092781 10.1200/JCO.2014.55.3628 PMC4162498

[18] Aplenc R Meshinchi S Sung L : Bortezomib with standard chemotherapy for children with acute myeloid leukemia does not improve treatment outcomes: A report from the Children's Oncology Group. Haematologica 105:1879-1886, 2020 32029509 10.3324/haematol.2019.220962 PMC7327649

[19] Creutzig U Zimmermann M Lehrnbecher T : Less toxicity by optimizing chemotherapy, but not by addition of granulocyte colony-stimulating factor in children and adolescents with acute myeloid leukemia: Results of AML-BFM 98. J Clin Oncol 24:4499-4506, 2006 16983120 10.1200/JCO.2006.06.5037

[20] Petit A Ducassou S Leblanc T : Maintenance therapy with interleukin-2 for childhood AML: Results of ELAM02 phase III randomized trial. Hemasphere 2:e159, 2018 31723797 10.1097/HS9.0000000000000159 PMC6745961

[21] van der Velden VHJ van der Sluijs-Geling A Gibson BES : Clinical significance of flowcytometric minimal residual disease detection in pediatric acute myeloid leukemia patients treated according to the DCOG ANLL97/MRC AML12 protocol. Leukemia 24:1599-1606, 2010 20668473 10.1038/leu.2010.153

[22] Waack K Schneider M Walter C : Improved outcome in pediatric AML—The AML-BFM 2012 study. Blood 136:12-14, 2020 (suppl 1)

[23] Tomizawa D Tawa A Watanabe T : Excess treatment reduction including anthracyclines results in higher incidence of relapse in core binding factor acute myeloid leukemia in children. Leukemia 27:2413-2416, 2013 23677335 10.1038/leu.2013.153

[24] Burnett AK Russell NH Hills RK : Optimization of chemotherapy for younger patients with acute myeloid leukemia: Results of the medical research council AML15 trial. J Clin Oncol 31:3360-3368, 2013 23940227 10.1200/JCO.2012.47.4874

[25] Lamble AJ Tasian SK: Opportunities for immunotherapy in childhood acute myeloid leukemia. Blood Adv 3:3750-3758, 2019 31770440 10.1182/bloodadvances.2019000357 PMC6880897

[26] Cooper TM Ries RE Alonzo TA : Revised risk stratification criteria for children with newly diagnosed acute myeloid leukemia: A report from the Children's Oncology Group. Blood 130:407, 2017

[27] Van Der Werf I Mondala P Diep R : Selective targeting of alternative splicing deregulation in pediatric acute myeloid leukemia stem and progenitor cells. Blood 136:8, 2020 (suppl 1)32614959

[28] Hanekamp D Denys B Kaspers GJL : Leukaemic stem cell load at diagnosis predicts the development of relapse in young acute myeloid leukaemia patients. Br J Haematol 183:512-516, 2018 29076143 10.1111/bjh.14991

[29] Witte KE Ahlers J Schafer I : High proportion of leukemic stem cells at diagnosis is correlated with unfavorable prognosis in childhood acute myeloid leukemia. Pediatr Hematol Oncol 28:91-99, 2011 21214408 10.3109/08880018.2010.528171

[30] Buldini B Maurer-Granofszky M Varotto E : Flow-cytometric monitoring of minimal residual disease in pediatric patients with acute myeloid leukemia: Recent advances and future strategies. Front Pediatr 7:412, 2019 31681710 10.3389/fped.2019.00412 PMC6798174

[31] Maurer-Granofszky M Schumich A Buldini B : An extensive quality control and quality assurance (QC/QA) program significantly improves inter-laboratory concordance rates of flow-cytometric minimal residual disease assessment in acute lymphoblastic leukemia: An I-BFM-FLOW-Network report. Cancers (Basel) 13:6148, 2021 34885257 10.3390/cancers13236148 PMC8656726

[32] Karol SE Coustan-Smith E Cao X : Prognostic factors in children with acute myeloid leukaemia and excellent response to remission induction therapy. Br J Haematol 168:94-101, 2015 25164427 10.1111/bjh.13107 PMC4262553

[33] Tomizawa D Tsujimoto SI Tanaka S : A phase III clinical trial evaluating efficacy and safety of minimal residual disease-based risk stratification for children with acute myeloid leukemia, incorporating a randomized study of gemtuzumab ozogamicin in combination with post-induction chemotherapy for non-low-risk patients (JPLSG-AML-20). Jpn J Clin Oncol 52:1225-1231, 2022 35809896 10.1093/jjco/hyac105

[34] Krivtsov AV Evans K Gadrey JY : A menin-MLL inhibitor induces specific chromatin changes and eradicates disease in models of MLL-rearranged leukemia. Cancer Cell 36:660-673.e11, 2019 31821784 10.1016/j.ccell.2019.11.001 PMC7227117

[35] Issa GC Aldoss I DiPersio JF : The menin inhibitor SNDX-5613 (revumenib) leads to durable responses in patients (pts) with KMT2A-rearranged or NPM1 mutant AML: Updated results of a phase (ph) 1 study. Blood 140:150-152, 2022 (suppl 1)

[36] Erba HP Fathi AT Issa GC : Update on a phase 1/2 first-in-human study of the menin-KMT2A (MLL) inhibitor ziftomenib (KO-539) in patients with relapsed or refractory acute myeloid leukemia. Blood 140:153-156, 2022 (suppl 1)

[37] Coustan-Smith E Campana D: Should evaluation for minimal residual disease be routine in acute myeloid leukemia? Curr Opin Hematol 20:86-92, 2013 23380603 10.1097/MOH.0b013e32835dd90a

